# Context and strain-dependent behavioral response to stress

**DOI:** 10.1186/1744-9081-4-23

**Published:** 2008-06-02

**Authors:** Katarzyna Nosek, Kristen Dennis, Brian M Andrus, Nasim Ahmadiyeh, Amber E Baum, Leah C Solberg Woods, Eva E Redei

**Affiliations:** 1 Northwestern University Feinberg School of Medicine, The Asher Center, Department of Psychiatry and Behavioral Sciences, Chicago, IL 60611, USA; 2 Brigham and Womens Hospital, 75 Francis St, and Dana-Farber Cancer Institute, 44 Binney St, Boston, MA 02115, USA; 3 National Institute of Health, 9000 Rockville Pike, Building 35, Room 1A207, Bethesda, MD 20892, USA; 4 Medical College of Wisconsin, 8701 Watertown Plank Road, CRI/TBRC Room C2415, Milwaukee, WI 53226, USA

## Abstract

**Background:**

This study posed the question whether strain differences in stress-reactivity lead to differential behavioral responses in two different tests of anxiety. Strain differences in anxiety-measures are known, but strain differences in the behavioral responses to acute prior stress are not well characterized.

**Methods:**

We studied male Fisher 344 (F344) and Wistar Kyoto (WKY) rats basally and immediately after one hour restraint stress. To distinguish between the effects of novelty and prior stress, we also investigated behavior after repeated exposure to the test chamber. Two behavioral tests were explored; the elevated plus maze (EPM) and the open field (OFT), both of which are thought to measure activity, exploration and anxiety-like behaviors. Additionally, rearing, a voluntary behavior, and grooming, a relatively automatic, stress-responsive stereotyped behavior were measured in both tests.

**Results:**

Prior exposure to the test environment increased anxiety-related measures regardless of prior stress, reflecting context-dependent learning process in both tests and strains. Activity decreased in response to repeated testing in both tests and both strains, but prior stress decreased activity only in the OFT which was reversed by repeated testing. Prior stress decreased anxiety-related measures in the EPM, only in F344s, while in the OFT, stress led to increased freezing mainly in WKYs.

**Conclusion:**

Data suggest that differences in stressfulness of these tests predict the behavior of the two strains of animals according to their stress-reactivity and coping style, but that repeated testing can overcome some of these differences.

## Background

The open field test is a complex behavioral paradigm, whose various component measures have been widely used to measure emotionality [1], exploration [2], general activity or locomotion [3], fear [3,4], and anxiety [5,6] in rodents. The elevated plus maze has also been widely used in rodents as a test of fear, anxiety [7], and more recently, risk assessment in mice [8,9] and rats [10,11]. On a pharmacological level, the OFT and EPM have both been validated as tests of anxiety [7,12].

Despite data supporting that OFT and EPM share areas of overlap in the behavioral traits they seek to measure and define, there are discrete but important differences between these two tests of anxiety. For instance, although activity in the OFT and open arm entries in the EPM share a genetic locus on chromosome 1, these same behaviors dissociate at the genetic locus found on chromosome 12 in mice [13]. Similarly, in rats, open arm entries in the EPM share a locus with rearing and activity in the OFT on chromosome 5, but not at other loci [14].

Factor analytic studies further illustrate that these tests measure different aspects of anxious behavior [15,16]. For example, OFT measures of rearing, outer line crossings, and inner line crossings all load unto a factor called general activity, while EPM measures of time spent in, and entries into open arms load onto an independent factor called anxiety [3]. In another study, only activity measures of OFT and EPM loaded into the same factor in a large F2 intercross of Roman rat strains [17].

Both the OFT and the EPM are inherently stressful tests, but to a different degree. If differences in the tests' stressfulness explain the non-overlapping measures in the OFT and EPM, repetition of these tests could eliminate these behavioral differences, and prior stress could accentuate them. Behavioral stress-responses are known to be altered by prior stress exposure depending on the nature of the prior stress, the stressfulness of the behavioral test and the individual variation in stress-responsiveness [18,19]. In this study, we compared the behavior of two inbred strains of rats, the Fisher 344 (F344) that is thought to be anxious and acutely hyper-reactive to stress [20-22] and the Wistar Kyoto (WKY) rats purported to be an endogenous model of chronic stress state and depression [23-30] in the OFT and the EPM.

## Methods

### Animals

Adult (12 weeks old) male WKY and F344 rats (Harlan Sprague Dawley, Indianapolis IN) were employed in these experiments. Animals were maintained at least two weeks prior the initiation of experiments in a controlled temperature vivarium on a cycle of 14 hours light, 10 hours darkness, and fed lab rat chow and water *ad libitum*. Animals were group-housed (3 per cage) at arrival, but individually housed for 5 days prior the beginning of the experiment. All behavioral tests were carried out on unhandled animals between 1000 and 1400 hr. Animals (n = 8–10/strain/group; 8 groups total) stayed in the stress room for two hours before testing began. On day 1 of the experiment, one group of animals was tested in one of two behavioral tests (OFT or EPM) in the adjacent testing room directly after being taken out of their home cage. A second group was tested immediately after a one hour period during which the animals were immobilized in a restraint apparatus. On day 2 the animals were exposed to the same treatments (restraint or no restraint) and the same tests (OFT or EPM) as on day 1. To avoid clues projected by the stressed animals, all non-stressed animals were tested before stressed animals and the stress room was cleaned thoroughly in between.

### Restraint

Minimal handling was used to insert rats into the restraint device, consisting of a close-ended transparent plastic cylinder. An adjustable insert was placed behind the body to secure the rat in the tube for an hour time period in a separate testing room. Large breathing holes at the front end of the tubes provided adequate ventilation.

### Open field test

The circular open field was constructed as previously described [30]. A wall of aluminum sheeting 30 cm high and painted dark gray surrounded an arena that was divided by three concentric circles of diameters 20, 50 and 82 cm respectively. The 50 cm-diameter circle defined the inner zone, the area between the 50 cm circle and the wall comprised the outer zone. The center zone was divided into 7 sections and the outer zone into 12 sections. The arena was lit from the ceiling of the room with incandescent lights, with a measurement inside the apparatus of 60 lux. The animal was placed in the 20 cm central circle and allowed to move freely for 10 minutes. A video camera was situated such that the entire field was visible, the test was video-taped, and the behavior was subsequently scored by two trained observers. The measures taken were number of lines crossed in the outer zone (outer lines), number of lines crossed in the inner zone (inner lines), total number of rears, duration of general grooming activity, latency to leave the center of the field (inner zone), and time spent in the center. The field was cleaned with a 1.25% acetic acid solution between trials to eliminate odor cues.

### Elevated plus maze

The maze was constructed as described by Pellow [7], with the following dimensions: central platform 10 × 10 cm, open arms 10 × 50 cm, closed arms 10 × 50 cm with a wall height of 40 cm, apparatus 50 cm above floor. The maze was lit from above with incandescent room light, showing a central platform light measurement of 60 lux. The animal was placed with its front paws on the center square facing a closed arm and allowed to move freely for 5 minutes. A video camera was used to record the test and was situated such that the entire maze, including the closed arms, was visible; the behavior was subsequently scored by two trained observers. The measures taken were: number of entries into open arms, number of entries into closed arms, number of total entries made, time spent in open arms, closed arms, in center, total number of rears and duration of general grooming activity. An arm entry was defined as all four paws in an arm. The maze was cleaned with a 1.25% acetic acid solution between trials to eliminate odor cues.

### Statistics

Data were analyzed by two-way ANOVA repeated measures design for day 1 and day 2 tests, with stress and strain as factors. The Bonferroni Multiple Comparisons test, with a p < 0.05 adjusted significance level, was used when appropriate to identify significant differences between groups.

## Results

### "Anxiety"-like behaviors

**Figure 1:**
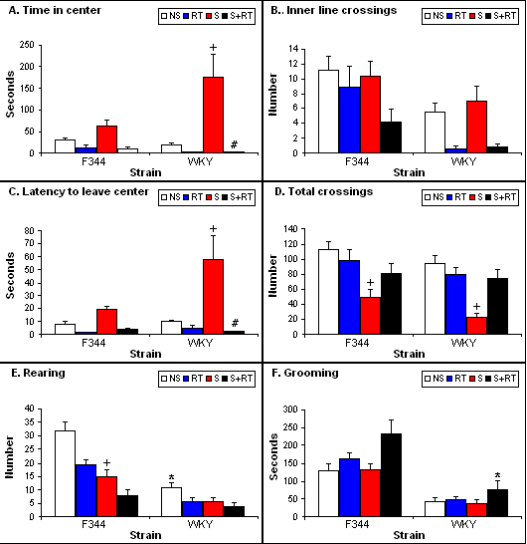
**Behavioral responses of adult male F344 and WKY in the OFT, basally (NS) and immediately after one hour restraint stress (S) without and with repeated testing (RT)**. (A) Time spent in center (seconds) was significantly increased by stress (S) in WKYs and reversed by repeated stress and test (S+RT); (B) inner line crossing; (C) latency to leave the center is significantly increased by stress in WKYs, and the effect is reversed by repeated testing; (D) number of total crossing was decreased by stress in both strains; (E) the number of rears differed significantly by strains in the NS group, and stress decreased number of rears significantly in F344; (F) time (seconds) spent grooming. Values are means +/- SEM. Asterisks indicate significant effect of strain; pound signs indicate significant effect of repeated testing; plus signs indicate significant effect of stress. All significant values are p < 0.05 by Bonferroni *post hoc *test.

There were no significant strain differences in the time F344s and WKYs spent in the center of the OFT (Figure 1a). Although stress increased time spent in the center of the OFT in both F344 and WKY strains, this increase was more profound in stressed WKYs (stress: F [1,71] = 15.2, p < 0.01; strain × stress: F = 6.89, p < 0.05). Test repetition decreased the amount of time spent in center in both strains, more so in the stressed animals, particularly WKYs (repeated testing: F = 28.94, p < 0.01; strain × repeated testing: F = 6.23, p < .05; stress × repeated testing: F = 15.90, p < 0.01; strain × stress × repeated testing: F = 6.15, p < 0.05).

Latency to leave the center of the OFT was generally higher in WKYs than in F344s (Figure 1c; strain: F = 8.33, p < 0.01). Stress prior to the test significantly increased latency in both F344 and WKY, but more so in WKYs (stress: F = 15.56, p < 0.01; strain × stress: F = 4.97, p < 0.05). Repeated testing decreased the latency to leave the center, and this effect is most obvious in stressed WKYs compared to stressed F344s (repeated testing: F = 30.53, p < 0.01; strain × repeated testing: F = 6.58, p < 0.05; stress × repeated testing: F = 16.50, p < 0.01; strain × stress × repeated testing: F = 8.13, p < 0.05).

The parallel pattern of changes in time spent in the center and latency to leave the center measures suggests that stress-induced increases in these parameters may not be related to decreased level of anxiety. Instead, the increased time in the center reflects freezing behavior in response to stress that is partially the result of the increased latency to leave the center.

A better measure of anxiety in the OFT seems to be the number of inner line crossings (Figure 1b). F344s crossed the inner circle significantly more than WKYs (strain: F = 14.45, p < 0.01). Stress prior to the tests had no effect on actively seeking the center, but repeated testing decreased the number of inner line crossings in both strains (repeated testing: F = 15.33, p < 0.01).

**Figure 2:**
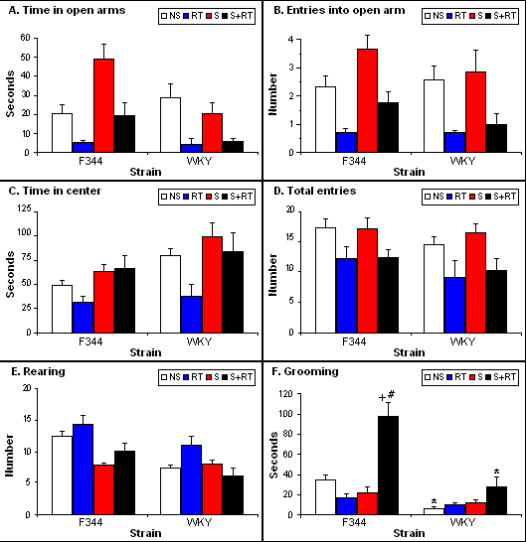
**Behavioral responses of the F344 and WKY in the EPM, basally and immediately after 1 hr restraint stress**. Groups are as described in Figure 1. (A) time spent (seconds) in open arms; (B) number of entries into open arms; (C) time spent (seconds) in center; (D) total number of entries (closed + open arm entries); (E) number of rears; (F) time spent (seconds) grooming. Values are means +/- SEM. Asterisks indicate significant effect of strain; pound signs indicate significant effect of repeated testing; plus signs indicate significant effect of stress. All significant values are p < 0.05 by Bonferroni *post hoc *test.

The classic anxiety measure, time spent in open arms of the EPM, did not differ between non-stressed F344s and WKYs (Figure 2a). Prior restraint stress resulted in increased time spent in the open arm, although this effect was seen only in F344s (strain × stress: F = 13.45, p < 0.01). Test repetition decreased time in the open arm regardless of stress and strain (repeated testing: F [1,88] = 13.74, p < 0.01).

The number of entries in the open arm of the EPM (Figure 2b) shows similar pattern as time spent in the open arm, indicating that these two measures likely reflect the animals' anxiety. The number of entries into the open arm was significantly affected by test repetition; it decreased the entries regardless of stress and strain (repeated testing: F = 16.90, p < 0.01).

Time spent in the center of the EPM was significantly higher in WKYs than in F344s (Figure 2c; strain: F = 6.77, p < 0.01). Stress increased time spent in the center in both F344s and WKYs (stress: F = 12.33, p < 0.01), while test repetition decreased it in animals not previously exposed to stress (repeated testing: F = 4.82, p < 0.05) in agreement with the other EPM anxiety measures.

### Activity

F344s were more active than WKYs in the OFT. Total number of crossings in the OFT were higher in F344s than in WKYs (Figure 1d; strain: F [1,72] = 4.71, p < 0.05). Stress decreased the total number of crossings in both F344s and in WKYs, however repeated testing post-stress reversed this effect (stress: F = 23.73, p < 0.01; stress × repeated testing: F = 12.00, p < 0.01).

Total number of entries in the EPM did not significantly differ between F344s and WKYs, and were not altered by prior stress (Figure 2d). In contrast, repeated testing decreased activity significantly in both strains (repeated testing: F1,86] = 10.5, p < 0.01).

### Activity/escape-orientated behavior

F344s reared more than WKYs in both tests (OFT, Figure 1e; strain: F [1,72] = 39.44, p < 0.01; EPM, Figure 2e; strain: F1,86] = 12.52, p < 0.01). In the OFT, test repetition decreased rearing (repeated testing: F = 12.13, p < 0.01), and prior stress had a similar effect, more so in F344s (stress: F = 21.42, p < 0.01; strain × stress: F = 7.91, p < 0.01). In contrast, test repetition slightly increased rearing in the EPM (repeated testing: F = 3.36, p = 0.06), but stress significantly decreased rearing, particularly in F344s, similarly to the effect in OFT (stress: F = 13.65, p < 0.01).

### Grooming

Fisher 344 spent significantly more time grooming than WKYs in both tests (OFT, Figure 1f; strain effect: F [1,72] = 53.49, p < 0.01; EPM, Figure 2f; strain: F [1,86] = 31.30, p < 0.01). In the OFT, test repetition increased grooming time of both the F344s and WKYs and this increase was more pronounced after prior stress (repeated testing: F = 8.33, p < 0.01). In contrast, animals groomed less after stress or after test repetition of the EPM, but stress and test repetition combined resulted in significantly increased time spent grooming (repeated testing: F = 13.69, p < 0.01; stress: F = 18.13, p < 0.01, stress × repeated testing: F = 24.35, p < 0.01), particularly of F344s (strain × stress × repeated testing: F = 15.94, p < 0.01).

## Discussion

This study posed the question whether strain differences in stress-reactivity lead to differential behavioral responses in two different tests of anxiety. The major findings of this study affirm this prediction. Restraint stress prior to the behavioral tests seems to have unmasked the differences: in the EPM, prior stress decreased anxiety-like behaviors only in F344s, while in the OFT, prior stress led to decreased activity in both strains. This decrease in activity was more pronounced in WKYs. These observations confirm the active coping style previously reported in F344s [31] and the passive coping style previously reported in WKYs [30-32]. Repeated testing resulted in an increase in anxiety in both tests and for both strains. Measures of activity decreased in response to repeated testing in both tests, but only in OFT in response to stress.

Repetition of the behavioral tests resulted in increased anxiety-related behavior in both tests and both strains of animals. Similarly, previous reports of repeated EPM testing found a reduction in open arm exploration [33-36], while others found no change from baseline [7,37,38]. Regarding what exactly occurs after repeated testing, no clear consensus is formed; whether familiarity causes a habituation or sensitization to fear via context-dependent learning, as it has been suggested previously [8,39,40], or whether repeated testing decreases the animal's novelty seeking behavior.

The differences between the F344 and WKY inbred strains in their anxiety-related behavioral response to stress are very prominent. Decreased anxiety is found in F344s by an increase in open arm exploration in the EPM. The unique profile of acute hyper-reactivity to stress seen in the F344 could be responsible for the stimulatory and anxiolytic effect of stress in this inbred strain. In contrast, Wistar Kyoto rats, with their purportedly chronic stress states responded minimally to prior acute stress in the EPM, but showed greater freezing than F344 rats in response to stress in the OFT. Thus, prior restraint stress in two strains previously shown to differ in stress reactivity, produced different behaviors in two different tests (OFT and EPM) long thought to measure some of the same aspects of behavior.

The paradoxical anxiolytic effects of stress on the F344s in the EPM, is confirmed by their grooming behavior in response to stress. Grooming is thought to be an indicator of stress perception and reactivity [41], and decreased grooming of F344s in the EPM post-stress, confirm their decreased stress perception or reactivity after stress. In contrast, grooming is dramatically increased in the second day tests in the stress group, suggesting that repeated testing induces sensitization to the test environment leading to learned avoidance as suggested before [39].

The reason for the differential effect of a prior stressor on F344s and WKYs in the EPM highlights their differences in reactivity to stress, mediated by the underlying genetic makeup of these two inbred strains. When stress is first applied, considerable excitation is observed, but if the stressor persists without successful coping, a longer period of behavioral depression follows with the animal behaving in a very passive fashion [42-45]. It has been suggested that the WKY progresses quickly through the activation phase of stress to the second phase of behavioral inhibition and passivity [23]. It seems likely, therefore, that our finding – that the F344 responds to stress in the EPM by increasing exploration of the open arms – reflects the propensity for the F344 to remain in the activating phase of stress for a longer time than the WKY, which, shortly after being faced with a stressor, rapidly enters the phase of passive coping. Likewise, our finding of increased open arm exploration after stress could reflect an increased sensitivity of the F344 to the activating effects of stress, manifesting itself as stress-induced increases in risk taking behaviors.

The differences in the behavioral responses recognized by the OFT and EPM may also be inherent in the design of these two tests. The OFT is aversive mainly due to factors of novelty whereas the EPM is aversive due to novelty as well as the height of the maze. In addition, the plus maze seems to offer more choices to animals when compared to the OFT, with the center of the plus maze being used as a crossroads or choice point, from where animals initially engage in high levels of risk assessment (see Rodgers [46], for review). Thus, if an animal is particularly indecisive or ambivalent about which arm to enter in the process of exploring the maze, that animal will likely spend more time in the center portion of the plus maze. In our study, the WKYs spend significantly more time in the center of the plus maze while spending less time in the closed arms of the maze than the F344s. We propose that this pattern of behavior in the WKYs can be viewed as ambivalence or indecision. Pare [29], in comparing WKYs to F344s in a modified one-way avoidance procedure, also found that the WKYs were more ambivalent than the F344s, and suggested that this was due to behavioral inhibition elicited by stressors used during the task. Since ambivalence or the inability to make decisions is frequently observed in clinical depression, our finding that the WKYs show increased ambivalence as measured by time spent in center of the plus maze further adds to the large body of work suggesting that the WKYs are in a state of chronic stress and reflect many characteristics of an animal model of depression [24,26,29,30,47,48].

## Conclusion

The present study shows that repeated testing increases anxiety-related measures in both strains regardless of prior stress. However, prior restraint stress exaggerates the differences between OFT and EPM, with stress resulting in a general decrease in behavioral responses in the OFT in both strains, contrasted with a strain-dependent response to stress in the EPM. Restraint stress results in decreased anxiety-like behavior (increased exploration of open arms) in the F344, with no change in the WKY, drawing attention to the importance of genetic differences of inbred strains in determining behavioral responsiveness to stress. Our original assumption that WKYs model at least some aspects of depressed behavior is supported by our finding of greater ambivalence, as measured by increased time spent in center of the plus maze, in the WKY as compared to the F344. The results of this study suggest that differences in the stressfulness of these tests may contribute to the distinction between them; the OFT is a better measure of passive coping while the EPM is a more sensitive measure of active coping in response to stress.

## Abbreviations

WKY: Wistar Kyoto; F344: Fisher 344; EPM: Elevated plus maze; OFT: Open field test.

## Competing interests

The authors declare that they have no competing interests.

## Authors' contributions

EER designed the study and the analysis, KN, KD, BMA, NA, AEB and LCSW conducted the behavioral experiments and scored the behaviors, KN, KD, BMA and EER analyzed the data and wrote the manuscript. All authors read and approved the final manuscript.
